# A Critical Review of Membrane Distillation Using Ceramic Membranes: Advances, Opportunities and Challenges

**DOI:** 10.3390/ma18143296

**Published:** 2025-07-12

**Authors:** Francesca Alessandro, Francesca Macedonio

**Affiliations:** Institute on Membrane Technology, National Research Council of Italy (CNR-ITM), via P. Bucci 17/C, 87036 Rende, CS, Italy; f.alessandro@itm.cnr.it

**Keywords:** membrane distillation, ceramic membranes, surface modification

## Abstract

Membrane distillation (MD) has attracted increasing attention as a thermally driven separation process for water purification, desalination, and wastewater treatment. Its primary advantages include high rejection of non-volatile solutes, compatibility with low-grade or waste heat sources, and operation at ambient pressure. Despite these benefits, large-scale implementation remains limited due to the lack of membrane materials capable of withstanding harsh operating conditions and maintaining their hydrophobic character. Polymeric membranes have traditionally been used in MD applications; however, their limited thermal and chemical stability compromises long-term performance and reliability. In contrast, ceramic membranes are emerging as a promising alternative, offering superior mechanical strength, chemical resistance, and thermal stability. Nevertheless, their broader adoption in MD is hindered by several challenges, including high thermal conductivity, surface wettability, high fabrication costs, and limited scalability. This review provides a critical assessment of current developments, key opportunities, and ongoing challenges associated with the use of ceramic membranes in MD. Particular emphasis is placed on advances in surface modification techniques and the emerging applications in advanced MD configurations.

## 1. Introduction

Water scarcity is a global issue exacerbated by rapid industrialization, urban development, and population growth [1]. To mitigate this problem, several water and wastewater treatment technologies have been developed [2], including adsorption [3,4], advanced oxidation process [5,6], reverse osmosis (RO) [7], catalysis [8,9], forward osmosis (FO) [10,11], and MD [12,13]. Despite significant advancements in water treatment technologies over the past two decades, the treatment of hypersaline wastewater—primarily brines from RO concentrate and mining operations—remains a major challenge due to their high salinity and complex chemical composition. Among the available technologies, MD has emerged as a promising solution for treating high-salinity streams due to its ability to be powered by low-grade heat [14], solar thermal energy [15], geothermal energy [16], or waste energy [17].

**Figure 1:**
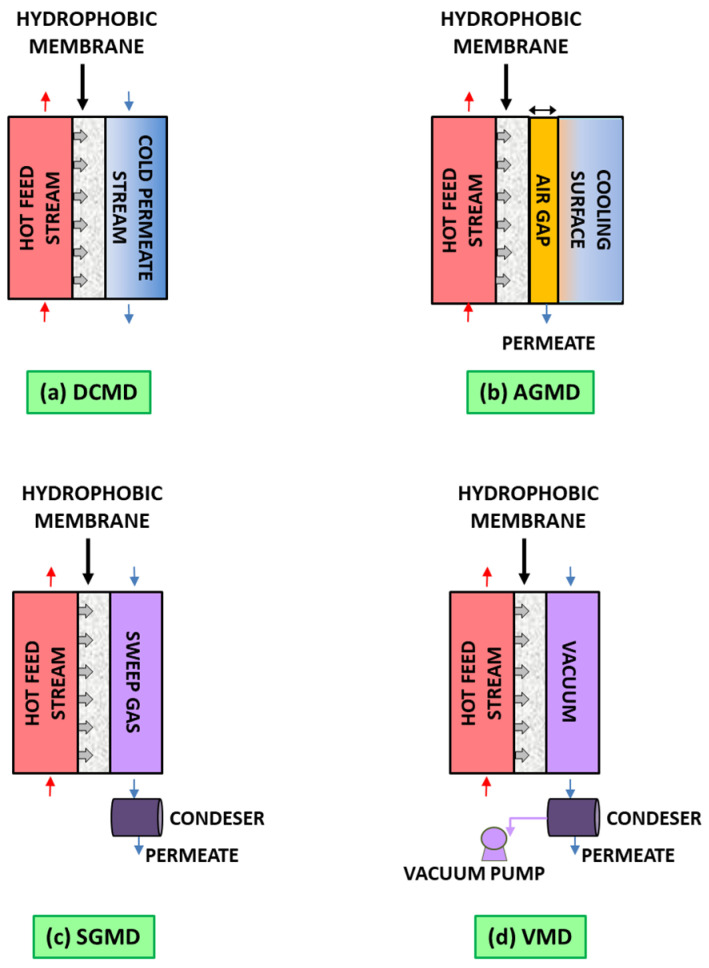
The four basic MD configurations: (**a**) direct contact membrane distillation (DCMD); (**b**) air gap membrane distillation (AGMD); (**c**) sweep gas membrane distillation (SGMD); and (**d**) vacuum membrane distillation (VMD).

Membrane distillation is a hybrid thermal–membrane separation process in which a temperature-induced vapor pressure gradient across a hydrophobic membrane drives the transport of volatile components—typically water vapor—while rejecting non-volatile solutes and contaminants [18,19]. When protracting the dehydration of the solution above the saturation limit, nucleation and crystal growth may occur. In this case, the process is referred to as membrane crystallization (MCr) [20], a technology that enables well-controlled crystallization by the manipulation of membrane physicochemical properties [21]. Mass transfer in MD occurs through Knudsen diffusion, molecular diffusion, and Poiseuille (viscous) flow, depending on the membrane pore size and the mean free path of the evaporated molecules [22]. On the other side, heat transport across the membrane occurs by two primary mechanisms: thermal conduction through the membrane material and latent heat transfer associated with the phase change of the vapor [23]. Depending on the way water vapor is condensed on the permeate side, MD can be classified into four basic configurations [24] (Figure 1): direct contact membrane distillation (DCMD), air gap membrane distillation (AGMD), sweeping gas membrane distillation (SGMD), and vacuum membrane distillation (VMD). Advantages and disadvantages related to each specific configuration are provided in Table 1.

**Figure 2:**
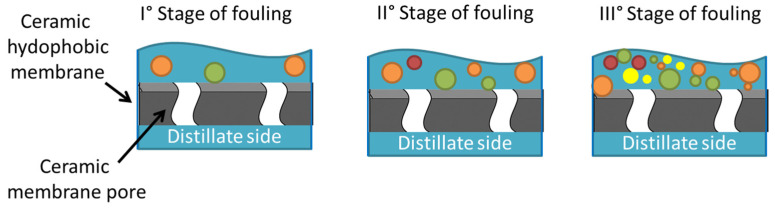
Schematic illustration of the progressive stages of fouling on a ceramic membrane surface. The first stage shows initial foulant deposition on the hydrophobic layer, followed by partial pore coverage in the middle stage, and finally complete pore blockage due to accumulated foulants in the final stage, compromising membrane performance.

Membrane distillation has been explored for desalination and wastewater treatment owing to its ability to operate at moderate temperatures (30–80 °C) and ambient pressure [25]. Compared to conventional pressure-driven membrane processes (such as RO, microfiltration (MF), ultrafiltration (UF), and nanofiltration (NF)), MD is less sensitive to fouling and concentration polarization, and it can theoretically achieve complete rejection of non-volatile compounds [26,27]. These properties make it particularly suitable for the treatment of highly saline and oily wastewaters, supporting the development of decentralized water treatment systems and zero-liquid discharge (ZLD) strategies [28]. Despite its potential, large-scale deployment of MD remains limited by several critical barriers, including membrane wetting and fouling, high energy consumption, low permeate flux, and limited long-term stability. Although the optimization of operating conditions and system design can improve flux and thermal efficiency, membrane fouling and wetting persist as critical obstacles [29]. Fouling reduces MD performance through the accumulation of contaminants on the membrane surface [30] (Figure 2).

Wetting, which occurs when the transmembrane pressure exceeds the liquid entry pressure (LEP), compromises overall system efficiency [31]. To mitigate these effects, membranes must exhibit strong anti-wetting properties, which are typically achieved by enhancing hydrophobicity and tailoring pore size and distribution [32,33]. In addition, membranes for MD application require high porosity, low thermal conductivity, and good mechanical, thermal, and chemical stability.

To date, polymeric membranes have been widely used in MD because of their intrinsic hydrophobicity, low surface energy, cost-effectiveness, and commercial availability [34,35]. Nevertheless, their limited thermal and mechanical stability under harsh operational conditions hinders their long-term application [35]. Recent investigations further revealed that PVDF membranes may undergo chemical interactions with lithium salts, particularly LiCl, leading to structural modifications in the polymeric matrix. These changes have been attributed to the coordination of Li^+^ ions with fluorine atoms in the PVDF chains, which can potentially alter the membrane hydrophobicity and long-term stability in lithium-rich environments [36]. In this context, ceramic membranes have attracted increasing attention [37,38] owing to their superior mechanical strength, as well as their excellent thermal and chemical stability, which contribute to enhanced operational durability. However, these membranes are inherently hydrophilic; therefore, surface modification is required to impart the hydrophobicity essential for MD applications [39,40]. Furthermore, their widespread adoption is constrained by high production costs, elevated thermal conductivity, and brittleness.

This review critically examines the current state of ceramic membranes for MD applications, with a focus on essential membrane properties, fabrication methods, and surface modification strategies. It also discusses key challenges and future directions aimed at enhancing membrane performance, lowering production costs, and enabling large-scale implementation, ultimately supporting the industrial adoption of ceramic membranes in advanced MD processes.

## 2. Ceramic Membrane Properties

### 2.1. Liquid Entry Pressure

As mentioned above, preventing membrane wetting is essential in MD systems, as mass transfer must occur exclusively through the vapor phase to ensure high rejection of non-volatile species and maintain process efficiency. This condition is met when the hydraulic pressure—used to recirculate the feed and permeate solutions within the module—remains below the LEP, which is defined by the Laplace equation [41]:

(1) $$LEP=-\frac{B\gamma_{l}cos\theta }{r_{max}}$$

where B is the pore geometric factor, $\gamma_{l}$ is the liquid surface tension (N/m), θ is the liquid–solid contact angle (◦), and $r_{max}$ is the maximum pore radius (m).

A minimum LEP of 250 kPa is generally recommended for MD operations [42], although reported values vary from 50 to 350 kPa for hydrophobic membranes and from 150 to 550 kPa for omniphobic membranes [43] (see Table 2). Notably, Hendren et al. [44] reported that ceramic membranes exhibit LEP values nearly twice as high as those of polymeric membranes. This significant difference is primarily attributed to variations in pore geometry and maximum pore size (r_max_) between the two membrane types.

Since LEP is influenced by several membrane characteristics—including maximum pore size, surface chemistry, and surface architecture—a thorough understanding of these parameters is critical for developing membranes with optimal performance for MD applications.

### 2.2. Pore Size, Porosity, and Thickness

The performance of MD systems is strongly influenced by key membrane properties such as porosity, pore size, tortuosity, and thickness. Lawson and Lloyd [45] proposed a relation that describes the molar flux through the membrane as a function of these parameters:

(2) $$N\propto \frac{\langle r^{\alpha }\rangle \cdot \varepsilon }{\tau \cdot \delta }$$

where ε is the membrane porosity, τ is the membrane tortuosity, δ is the membrane thickness, and $\langle r^{\alpha }\rangle$ is the mean pore radius, with α=1 for Knudsen diffusion and α=2 for viscous flow regimes.

Therefore, to achieve high permeability, the surface layer that controls the membrane transport must be as thin as possible $\left(\tau \right)$, whereas porosity and the pore size should be as large as possible. However, it is important to note that an increase in pore size also leads to a reduction in the LEP, as described by Equation (1), which may compromise the membrane wetting resistance. According to Table 2, ceramic membranes used in MD applications typically exhibit pore sizes ranging from 0.1 to 1 μm and porosity between 25% and 60%. These values are generally lower than those of polymeric membranes, which often exceed 70% porosity. Because high porosity increases the effective evaporation area and thereby enhances permeate flux, further optimization of ceramic membrane porosity is necessary to improve MD performance.

**Table 2:** Properties of hydrophobic ceramic membrane used in MD application. Data extracted from [46,47,48,49,50,51,52,53,54,55,56,57,58,59,60].

Year	Membrane Material	Membrane Configuration	Pore Size (μm)	Porosity (%)	Thickness (μm)	Contact Angle (°)	LEP (kPa)	Ref.
2004	Alumina	Tubular	0.2	N.A.	N.A. *	123–143	N.A.	[46]
	Zirconia		0.05	N.A.	N.A.	116–145	N.A.	
2011	Tunisian clay	Tubular	0.18	N.A.	35	177–179	N.A.	[47]
2014	Titania	Tubular	0.170–0.175	N.A.	N.A.	135–145	300–900	[48]
2015	Alumina	Flat sheet	0.5–1	59	780	133	200	[49]
2016	Almina	Tubular	0.02–0.04	30	N.A.	120	100–500	[50]
	Titania	Flat sheet	0.2	40	N.A.	148	N.A.	
2016	Alumina	Flat sheet	1–2	62	45	135–137	220–230	[51]
2016	β-sialon	Hollow fiber	0.80	45–60	250	125	310	[52]
2018	Alumina	Tubular	0.333	23.3	200	138	N.A.	[53]
	Alumina	Hollow fiber	0.22	55	200	137	N.A.	
2018	Alumina	Hollow fiber	N.A.	N.A.	N.A.	140	235	[54]
2019	Alumina/ zinc oxide	Hollow fiber	0.90	26.64	201	134	110	[55]
2021	Mullite-kaolite	Hollow fiber	N.A.	10.8	223	49.5	N.A.	[56]
2021	Palm oil fuel shell	Hollow fiber	1.05	39.2	N.A.	149	289.6	[57]
2022	Ball clay	Hollow fiber	0.1–1.2	N.A.	N.A.	>150	33–133	[58]
2023	Coal fly ash	Tubular	0.18	N.A.	40–100	123.1–125.5	>60	[59]
2025	Wollastonite	Planar	0.51	27.70	N.A.	150	125	[60]

* N.A.: Not Available.

Membrane thickness also plays a crucial role. Reducing membrane thickness decreases resistance to vapor transport and enhances transmembrane flux. However, increasing membrane thickness improves thermal insulation by limiting conductive heat loss. Considering this trade-off, the optimal membrane thickness for MD operations is typically between 30 and 60 μm [61]. In contrast, ceramic membranes often exhibit wall thicknesses of up to 200 μm [42], which can significantly reduce both mass transfer rates and thermal efficiency.

### 2.3. Thermal Conductivity

In MD, minimizing thermal conductivity is crucial to reduce conductive heat losses, alleviate temperature polarization, and sustain a high vapor pressure gradient across the membrane. Polymeric membranes—such as polypropylene (PP), polyvinylidene fluoride (PVDF), and polytetrafluoroethylene (PTFE)—commonly used in MD, exhibit inherently low thermal conductivities, typically ranging from 0.19 to 0.27 W·m^−1^·K^−1^ for PP, 0.25 to 0.27 W·m^−1^·K^−1^ for PVDF, and 0.11 to 0.16 W·m^−1^·K^−1^ for PTFE [62]. These values contribute to reduced conductive heat transfer and improved thermal insulation, which are favorable for MD performance. Conversely, ceramic membranes, while offering superior chemical and thermal stability, generally exhibit significantly higher thermal conductivities. This intrinsic property limits their thermal efficiency in MD applications. Consequently, recent research has focused on developing novel ceramic materials and membrane architectures aimed at reducing thermal conductivity without compromising and potentially enhancing separation efficiency. Yang et al. [63] synthesized γ-Y_2_Si_2_O_7_ flat-sheet membranes exhibiting thermal conductivities as low as 0.497 W·m^−1^·K^−1^ at 32 °C and 0.528 W·m^−1^·K^−1^ at 100 °C. These membranes achieved a water flux of 10.07 kg·m^−2^·h^−1^ and salt rejection greater than 99.9% using 20 wt% NaCl feed solution at 90 °C. In another study, hydrophobic zirconia-based nanofiber membranes demonstrated an ultra-low thermal conductivity of 0.044 W·m^−1^·K^−1^ [62], significantly lower than that of conventional polymeric MD membranes. This reduction in conductive heat loss resulted in a high permeate flux (32.1 LMH) and improved thermal efficiency, as confirmed through both experimental testing and computational fluid dynamics (CFD) simulations [64]. These advancements highlight the potential of low-thermal-conductivity ceramic materials to improve MD performance and pave the way for high-efficiency membranes suitable for industrial-scale applications.

### 2.4. Chemical and Thermal Stability

The chemical stability of membrane materials is a critical factor in determining long-term performance, particularly under aggressive operating conditions. Chemical interactions between the membrane and the feed solution can alter membrane structure, compromise surface properties, and reduce separation efficiency. In particular, feed solutions containing acid gases or oxidizing agents are especially corrosive and can lead to rapid membrane degradation. Ceramic membranes demonstrate exceptional chemical stability across a wide range of pH and temperature conditions. Unlike polymeric membranes—which are prone to degradation, pore collapse, and loss of hydrophobicity when exposed to heat or corrosive media—ceramic materials such as alumina, zirconia, and silicon carbide retain their structural integrity and functional properties even in extreme environments. For instance, Boey et al. [64] developed a flexible hydrophobic zirconia-based ceramic nanofibrous membrane that demonstrated remarkable chemical stability in highly corrosive environments. After immersion in both acidic (0.1 M HCl) and alkaline (0.1 M NaOH) solutions for 24 h, the membrane maintained its structural integrity, stable permeate flux, and high salt rejection rates (>99%) during MD tests. These findings not only highlight the excellent chemical resilience of zirconia-based ceramic membranes but also underscore the broader potential of ceramic materials to preserve their structural and functional integrity under extreme chemical and thermal conditions. Their resistance to both acidic and alkaline environments suggests suitability for treating industrial hypersaline brines, which typically contain high salt concentrations and various corrosive species, making ceramic membranes particularly attractive for demanding MD applications such as industrial wastewater treatment and brine concentration. By maintaining stable performance under both thermal and chemical stress, ceramic membranes offer a clear advantage over polymeric alternatives in terms of operational lifespan, reliability, and suitability for harsh MD conditions.

### 2.5. Membrane Configuration

The industrial deployment of MD requires hydrophobic membranes with large surface areas to achieve high water production rates. Therefore, optimizing membrane configuration and module design plays a pivotal role in improving system performance, operational stability, and cost-effectiveness.

**Figure 3:**
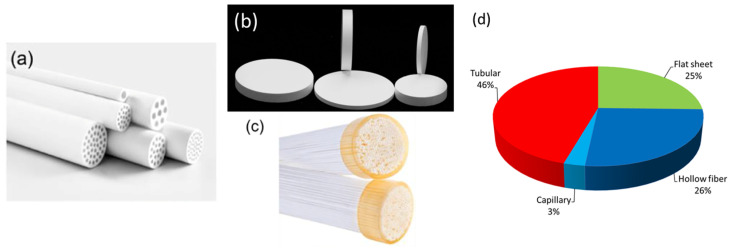
(**a**) Tubular ceramic membrane (Jiangsu Jiuwu HI-TECH Co., Ltd., Nanjing, China), (**b**) disc ceramic membrane (Cobra Technologies BV, Rijssen, The Netherlands), (**c**) hollow fiber ceramic membrane (Hyflux Ltd., Singapore), (**d**) membrane configurations of ceramic membranes used in MD. (Data adapted from Scopus from 2004 to December 2024).

Ceramic membranes for MD are typically fabricated in three main configurations: planar, tubular, and hollow fiber (Figure 3a–c). Each configuration offers specific advantages and limitations in terms of mechanical strength, packing density, and scalability. Ceramic planar membranes are usually produced in the form of flat sheets or discs and are often integrated into plate-and-frame modules. They are among the most widely manufactured configurations, alongside tubular ceramic membranes (Figure 3d). Planar membranes offer several advantages, such as ease of cleaning, simple replacement, and uniform flow distribution. These membranes are generally fabricated via slip casting, tape casting, or pressing techniques that enable precise control over membrane thickness and porosity. However, planar modules are limited by relatively low packing densities (100–400 m^2^/m^3^) and reduced thermal efficiency, which constrain their application in large-scale MD systems [65].

Tubular membranes, typically produced by extrusion, provide superior mechanical strength and robustness during operation. Although they offer better mechanical stability than flat membranes, their packing density is also relatively low (below 300 m^2^/m^3^), which translates into higher capital and operational costs per unit of water produced [66].

Hollow fiber membranes represent a major advancement in MD membrane design. With diameters in the sub-millimeter range and an asymmetric structure that favors vapor transport, these membranes achieve extremely high surface-area-to-volume ratios. Fabricated using phase inversion-based extrusion, hollow fiber membranes can reach packing densities up to 9000 m^2^/m^3^, making them ideal for compact MD modules [66,67]. However, their small internal diameters make them prone to fouling and clogging, and their relatively low mechanical strength poses a risk for industrial operation.

To address these limitations, multichannel monolithic ceramic membranes have recently been developed. These advanced structures integrate multiple flow channels within a single monolithic block, improving both specific surface area and mechanical durability. In addition, they exhibit enhanced antifouling performance due to optimized hydrodynamics and channel geometry [54]. As a result, multichannel membranes represent a promising direction for scalable, high-performance MD systems suitable for industrial applications.

## 3. Surface Modification of Ceramic Membranes: From Intrinsic Hydrophilic to Functional Hydrophobic

**Figure 4:**
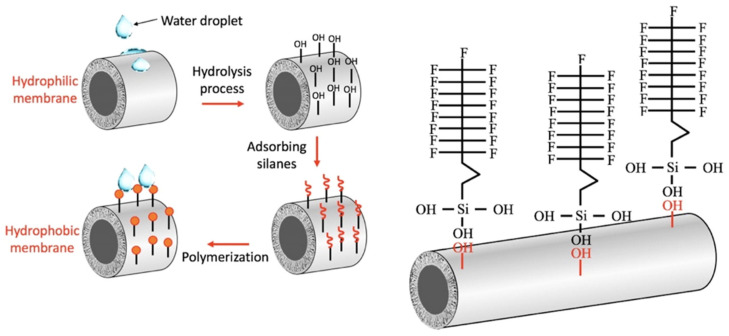
Schematic representation of the surface modification mechanism of ceramic membranes using organosilanes. Copyright 2022 Elsevier. Reprinted from Ref. [69] (open access).

As discussed in Section 2, compared to polymeric membranes, ceramic membranes offer superior thermal and chemical stability and greater mechanical strength, making them attractive candidates for MD applications. However, ceramic membranes exhibit intrinsic hydrophilicity due to the presence of surface hydroxyl (–OH) groups, which readily form hydrogen bonds with water molecules. While this property facilitates water transport in conventional filtration processes, it poses a significant drawback in MD, where hydrophobicity is essential to prevent liquid intrusion into the membrane pores. Therefore, achieving high LEP and sustained wetting resistance is are critical prerequisite for stable MD operation. To overcome the hydrophilic nature of ceramic materials, various surface modification techniques (such as chemical grafting, physical deposition, and fluorine-free coating) have been developed to tailor membrane wettability [39,40,41]. These techniques vary in durability, scalability, ease of implementation, and environmental impact, as summarized in Table 3. Among them, chemical grafting with organosilanes—particularly fluoroalkylsilanes (FASs) such as PFOTES and methyltrichlorosilane (MTS)—is one of the most widely adopted approaches. Silanes covalently bond to surface –OH groups via condensation reactions, forming stable Si–O–M (M = metal) linkages that yield durable hydrophobic surfaces (Figure 4) [68,69]. Optimized silanization protocols typically employ silane concentrations of 1–10 wt%, reaction times of 12–72 h, and curing temperatures between 70 and 150 °C, resulting in superhydrophobic surfaces with water contact angles (WCAs) exceeding 150°. For instance, Chen et al. [70] modified tubular alumina membranes using hexadecyltrimethoxysilane (C16), achieving WCAs above 150°. The modified membranes demonstrated stable performance over 1000 min of continuous VMD operation, with permeate fluxes around 30 kg·m^−2^·h^−1^ and salt rejection of approximately 99.9%.

An effective strategy to further enhance surface hydrophobicity and prolong wetting resistance is the development of hierarchical surface structures. These micro/nano-scale textures promote the Cassie–Baxter wetting regime by trapping air pockets within surface asperities, thereby preserving superhydrophobicity even as low-surface-energy coatings degrade. Hierarchical structuring is typically achieved via hydrothermal synthesis of inorganic nanostructures such as ZnO nanorods, TiO_2_ microflowers, and Al_2_O_3_ nanoflowers [71,72,73].

**Figure 5:**
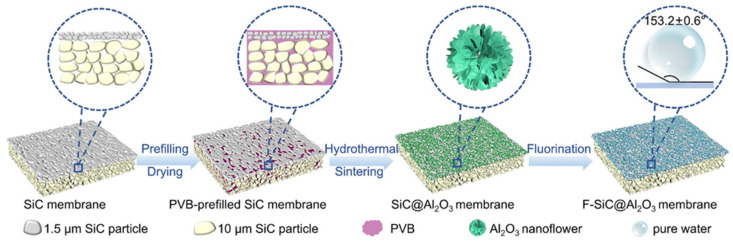
Scheme of the in situ growth of Al_2_O_3_ nanoflowers. Reprinted with permission from Ref. [74]. Copyright 2025 Elsevier.

For example, Miao et al. [74] developed Al_2_O_3_-coated ceramic membranes through in situ hydrothermal growth of nanoflowers on SiC supports, followed by fluorination (Figure 5). To avoid pore blockage, the substrates were prefilled with 3 wt% polyvinyl butyral (PVB), allowing uniform formation of ~1.2 μm Al_2_O_3_ structures. The resulting membranes exhibited increased surface roughness (1.04 → 3.08 μm), reduced pore size (0.635 → 0.214 μm), and superhydrophobicity (WCA = 153.2 ± 0.6°). In VMD tests with NaCl solutions, these membranes delivered stable fluxes up to 13.2 kg·m^−2^·h^−1^ and salt rejection exceeding 99.9%, marking an 81.6% improvement over unmodified membranes. Similarly, Omar et al. [75] applied a two-step modification involving hydrothermal deposition of CuO flakes followed by octyltrichlorosilane (C8) grafting on mullite–kaolinite membranes. The resulting surfaces achieved WCAs of 155.9° and LEP values up to 5.2 bar. To improve scalability and ensure uniform coatings, physical deposition techniques such as electrospraying and spray-coating have also been investigated. These methods enable the application of polymer–nanoparticle composites (e.g., PVDF-HFP/ZnO) onto ceramic substrates, offering tunable layer thicknesses, enhanced adhesion, and better control over surface morphology [58]. Compared to traditional immersion techniques, membranes fabricated via spray-coating have demonstrated superior wetting resistance and mechanical robustness, particularly under cyclic MD conditions [58].

Nonetheless, the widespread use of fluorinated silanes raises environmental concerns due to their persistence and potential toxicity. As a result, growing attention has been directed toward fluorine-free hydrophobization strategies. Fluorine-free hydrophobic coatings represent a promising direction to address both environmental and regulatory challenges associated with conventional fluorinated silanes. While traditional fluorinated compounds (e.g., PFOTES) offer excellent hydrophobicity (WCA > 150°), they pose serious concerns due to their high environmental persistence, bioaccumulation, and potential toxicity. Moreover, increasing global regulatory pressure—such as the EU REACH regulation and national restrictions on per- and polyfluoroalkyl substances—is accelerating the transition toward fluorine-free alternatives.

Recent studies have demonstrated that fluorine-free coatings can achieve comparable wetting resistance and long-term stability. For instance, Lv et al. [60] fabricated wollastonite membranes coated with methylphenyl silicone resin, achieving WCA > 150° and stable VMD operation for over 120 h with salt rejection rates above 99.9%. Similarly, boron nitride (BN) nanosheets have enabled superhydrophobic surfaces with WCAs up to 130–145°, exhibiting excellent chemical and thermal resistance even under high-salinity and high-temperature conditions [76,77]. A β-sialon membrane coated with BN nanosheets was fabricated via chemical vapor deposition (CVD), showing stable MD performance with >99.9% salt rejection and consistent flux over 200 h, highlighting its potential for robust long-term desalination applications [76].

From a mechanical perspective, fluorine-free coatings often show improved adhesion to ceramic substrates, reducing the risks of delamination during cyclic MD operations. In addition, they provide greater compatibility with hierarchical micro/nano-structured surfaces, enhancing robustness against mechanical and chemical degradation [78]. These fluorine-free coatings contribute to improving the environmental compatibility and overall sustainability of ceramic MD membranes, aligning with global efforts to reduce the use of persistent and potentially harmful substances.

In addition to traditional structural design methods, recent advances in additive manufacturing (3D printing) have opened new possibilities for engineering ceramic membrane architectures with tailored geometries and hierarchical porosity [79,80]. Unlike conventional fabrication techniques, 3D printing allows precise control over pore size distribution, channel configuration, and overall three-dimensional architecture [81]. This approach enables the fabrication of complex lattice-like or multichannel structures, which can significantly enhance mass and heat transfer, reduce temperature and concentration polarization, and improve mechanical stability [82]. Furthermore, the design flexibility and material tunability offered by 3D printing facilitate the integration of surface modifications or functional coatings (e.g., hydrophobic or photothermal layers) directly onto the membrane structure. Among the various additive manufacturing techniques, direct ink writing (DIW), fused deposition modeling (FDM), digital light processing (DLP), and inkjet 3D printing have all been explored for water treatment applications. DIW and FDM are particularly suitable for ceramic-based structures due to their compatibility with highly viscous ceramic inks and thermoplastic feedstocks, enabling robust and complex porous architectures. In contrast, DLP provides higher resolution but requires photoactive resins, making it less versatile for purely ceramic materials. Inkjet 3D printing, while offering precise layer control, is mainly used for thin coatings or functional surface modifications rather than bulk ceramic structures. The choice of technique ultimately depends on the desired resolution, material compatibility, and mechanical requirements of the final membrane [83]. Despite current limitations such as high fabrication costs and the need for post-processing (e.g., sintering), 3D-printed ceramic membranes represent a promising strategy for the next generation of MD applications. They offer opportunities for integrated mechanical reinforcement and application-specific design, supporting the development of more efficient and resilient water treatment systems.

Therefore, combining advanced surface chemistry modification with innovative structural design approaches enables the development of ceramic membranes that exhibit high hydrophobicity, mechanical robustness, and superior operational stability, which are critical for the successful implementation of MD technologies.

## 4. Ceramic Membranes in MD Applications

**Figure 6:**
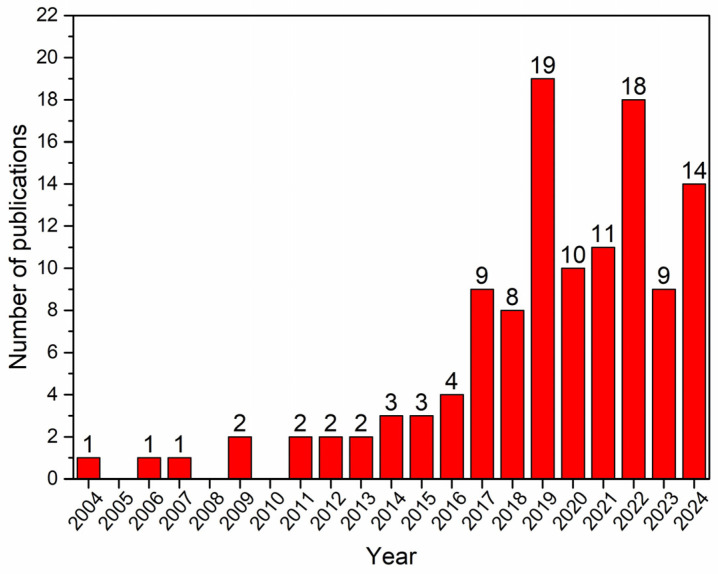
Number of publications on ceramic membranes for MD applications. (Data adapted from Scopus from 2004 to December 2024).

The first use of ceramic membranes in MD was reported by Larbot et al. [46]; in their study, hollow fiber alumina and zirconia membranes were grafted with fluoroalkylsilanes to impart hydrophobicity to the surface. The modified membranes were tested in DCMD desalination, demonstrating excellent salt rejection (100%) and water vapor fluxes ranging from 0.5 to 8.43 L·m^−2^·h^−1^ [46]. Since then, interest in ceramic membranes for MD has grown significantly, as reflected in the increasing number of publications over the years (Figure 6). As summarized in Table 4, most studies have focused on desalination, with ceramic membranes applied across all basic MD configurations. These membranes typically exhibit stable performance and high rejection efficiencies—often exceeding 99.9%—across a broad range of saline feedwaters. For example, Alftessi et al. [69] developed a porous hollow fiber ceramic membrane using low-cost silica sand and functionalized it with fluoroalkylsilane (FAS17). When tested in DCMD with 0.8 wt% NaCl over 32 h, the membrane achieved a vapor flux of 35 kg·m^−2^·h^−1^ and complete salt rejection.

Although most studies have highlighted ceramic membranes for desalination, few studies have explored their use in the removal of specific inorganic contaminants. Hubadillah et al. [84] prepared ceramic hollow fiber membranes from rice husk ash to remove arsenic species (As(III) and As(V)) via DCMD. Membranes prepared from amorphous (ASHFM) and crystalline (CSHFM) silica were modified with fluoroalkylsilanes, achieving superhydrophobicity with contact angles of 157° and 161°, respectively. The CSHFM membrane showed superior performance, with vapor fluxes of 50.4 and 51.3 kg·m^−2^·h^−1^ for As(III) and As(V), while arsenic rejection reached at most 99.6% [84].

**Table 4:** MD performance for ceramic membrane reported in the literature.

No.	Year	Material	Grafting Agent	WCA (°)	Membrane Configuration	MD Configuration	Application	Feed Solution	Feed Temp (°C)	Permeate Side	Permeate Flux (Lm^2^h^−1^)	Rejection (%)	Ref.
1	2004	Zirconia/alumina	1H,1H,2H,2H-Perfluorodecyltriehoxysilane (PFDTES)	145	Tubular	DCMD	Desalination	0.001–2.9 M	60–95	5 °C	0.87–5.4	~100	[46]
2	2006	Zirconia/alumina	PFDTES	N.A. *	Tubular	AGMD	Desalination	1 M NaCl	95	5 °C	5.42	100	[85]
3	2007	Zirconia	Tridecafluoro-1,1,2,2-tetrahydrooctyltriethoxysilane (T-PES)	N.A	Tubular	AGMD	Desalination	0.5–2 M NaCl	95	5 °C	12.62	>95	[86]
4	2009	Zirconia	T-PES	160	Tubular	VMD/AGMD/DCMD	Desalination	0.5–1 M NaCl	75–95	Vacuum 3 mbar/5 °C	7.5/4.7/4	99.8/99.8/96.1	[87]
5	2011	Tunisian clay	FAS	180	Planar	AGMD	Desalination	1 M NaCl	75–95	5 °C	3.2–6.45	99	[47]
6	2014	Silicon nitride	Tridecafluoro-1,1,2,2-tetrahydrooctyltriethoxysilane (T-PFOS)	136	Hollow fiber	VMD	Desalination	0.5, 2, 4, 6 wt% NaCl	50–80	0.02 bar	15.1–22.25	99–100	[88]
7	2014	Titania	FAS	132	Tubular	AGMD	Desalination	0.5 M NaCl	90	5 °C	1.2	N.A.	[89]
8	2015	Alumina	T-PFOS	133	Planar	DCMD	Desalination	2 wt% NaCl	80	20 °C	19.1	99.5	[49]
9	2016	β-sialon	T-PFOS	125	Hollow fiber	DCMD/VMD	Desalination	2 wt% 4 wt%	50–80	20 °C/0.02 bar	7.92–10.75	99–100	[52]
10	2016	Clay-alumina	Tridecafluoro-1,1,2,2-tetrahydrooctyltrimethoxysilane (T-PFS)	145	Capillary	AGMD	Desalination	0.5 M NaCl	60	feed pressure of 0.85 bar	4.1	99.96	[90]
11	2017	β-sialon	PDMS	145	Flat sheet	SGDM	Desalination	4 wt% NaCl	90	N.A.	14.08	>99	[91]
12	2017	Alumina	T-PFS	137	Hollow fiber	VMD	Desalination	3.5 wt% NaCl	70	0.03 bar	60	>9.9	[92]
13	2018	Silica/alumina	T-PFOS	158	Hollow fiber	VMD	Desalination	3.5 wt% NaCl	70	98 kPa	29.3	99.9	[93]
14	2018	Rice husk ash	T-PFS	>150	Hollow fiber	DCMD	Desalination	3.5 wt% NaCl	65	25 °C	38.20	>99.9	[94]
15	2019	Yttrium	Dimethyldichlorosilane (DMDCS)/dichloromethy-Isilane	132	Flat sheet	SGMD	Desalination	20 wt% NaCl	90	5 °C	10.07	99.9	[63]
16	2019	Kaolin	FAS	145	Hollow fiber	DCMD	Arsenic removal	As(V)	60	15 °C	28	100	[95]
17	2020	Alumina	Methyltrichlorosilane (MTCS)	145	Tubular	VMD	Desalination	9 wt% NaCl	70	20 mbar	31.2	100	[96]
18	2020	Alumina/Titania	1H,1H,2H,2H-perfluorodecylsilane-triethoxy (PDTS)	152	Hollow fiber	VMD	Desaliation	10 wt% NaCl	65	0.085 kPa	5.81	100	[97]
19	2020	Kaolin	FAS	146	Hollow fiber	DCMD	Arsenic removal	As	60	10 °C	23.3	100	[98]
20	2021	Alumina, titania, cordierite	Tridecafluoro-1,1,2,2,-tetrahydrooctyltriethoxysilane (TFTES)	N.A	Tubular	DCMD	ZLD	30 g NaCl/kg H_2_O	60	20 °C	25	>99.9	[99]
21	2021	Red clay	N.A.	95.4	Tubular	VMD	Desalination	1 wt% NaCl	70	3.5 mbar	13.10	98.96	[100]
22	2022	Ball clay	ZnO nanoparticles with T-PFOS	>150	Hollow fiber	DCMD	Desalination	1 wt% NaCl	80	10 °C	6.2	>99.8	[72]
23	2022	Cenosphere	Poly(dimethyl siloxane)	119.2	N.A.	DCMD	Desalination	2 wt% NaCl	90	5 °C	13	99	[101]
24	2022	β-sialon	Boron nitride	145	Planar	SGMD	Desalination	2 wt% NaCl	80	Nitrogen	7.7	99.9	[76]
25	2022	Metakaolin	1H,1H,2H,2H-perfluorooctyltriethoxysilane (FOTS)	143.3	Flat sheet	DCMD	Desalination	3.5 wt% NaCl	80	Flow rate of 40 L/h	6.58	>95	[102]
26	2022	Si_2_N_2_O	Dichloromethylsilane (DMDCS)	152	Planar	SGMD	Desalination	4 wt% NaCl	70	Dry air	15.6	>99.9	[103]
27	2022	Silica sand	1H,1H,2H,2H-Perfluorodecyltriethoxysilane (FAS17)	142.5	Hollow fiber	VMD	Desalination	0.8 wt% NaCl	80	25 °C water	35	100	[69]
28	2023	Al_2_O_3_/TiO_2_	C16	143.3–144.4	Tubular	PVMD	Desalination	Produced water (TDS di 135 g/L, TDS di 175 g/L)	68.5	vacuum 11 kPa	2.88–3.02	99.9	[104]
29	2023	Al_2_O_3_/TiO_2_	Alkyl triethoxysilane	140	Tubular	VMD	ZLD	~26 wt% NaCl	75	vacuum 7.5 kPa	35	99.98	[105]
30	2023	Coal fly ash	1H,1H,2H,2H-Perfluorodecyltrichlorosilane (PFDTS)	>123	Tubular	VMD	Desalination	1 wt% NaCl	55	vacuum 40 kPa	9.54	98.36	[59]
32	2024	Kaolin	1H,1H,2H,2H-Perfluorododecyltrichlorosilane (PFDDoTS)	160	Flat-sheet	AGMD	Desalination	7 wt% NaCl	70	Air gap/20 °C cold	7/9.3	>99.99	[106]
33	2024	Mullite	Hexadecyltrimethoxysilane (HDTMS)	160	Tubular	DCMD	Desalination	3.5 wt% NaCl	68	15	3.15	99.6	[107]
34	2024	SIC/alumina	PFDTES	152.4	Disc	VMD	Desalination	3.5 wt% NaCl	70	vacuum 90 kPa	11.1	99.9	[73]
35	2024	Mullite/kaolin	PFDTES	155.9	Hollow fiber	DCMD	Desalination	3.5 wt% NaCl	70	10 °C water	24.3	99.91	[75]
36	2024	Wollastonite	Methylphenyl Silicone Resin	150	Planar	VMD	Desalination	3.5 wt% NaCl	80	vacuum 100 kPa	35.2	99.9	[60]
37	2025	Cordierite clay	PFDTES	145.5	Capillary	AGMD	Desalination	3.5 wt% NaCl	85	Air gap/5 °C cold	3	95.0	[108]

* N.A.: Not Available.

Recent advances in fabricating hydrophobic ceramic membranes from low-cost or renewable sources—such as rice husk ash—highlight their promise for sustainable and efficient MD-based treatment of contaminated water. Their successful use in non-traditional applications, including heavy metal removal and high-salinity brine concentration, underscores the versatility of ceramic membranes. Nevertheless, key challenges remain. Long-term operational stability under real wastewater conditions, membrane fouling behavior, and the scalability of membrane fabrication must be addressed before ceramic MD membranes can be widely deployed in decentralized and industrial water treatment systems.

## 5. Critical Challenges and Emerging Strategies for Ceramic Membranes Development in MD

### 5.1. High Fabrication Cost

The widespread adoption of ceramic membranes in the MD process remains limited by their elevated fabrication costs. While the market prices of conventional ceramic oxides, such as alumina (480–550 USD/ton) and zirconia (1500–2000 USD/ton), are comparable to those of polymeric materials, like polyethylene terephthalate (PET, 1000–2000 USD/ton), the overall production costs of ceramic membranes are significantly higher [109].

This disparity is primarily attributed to the complexity of ceramic membrane fabrication, which typically involves high-temperature sintering and precise structural control. Furthermore, the inherently hydrophilic nature of ceramic surfaces necessitates additional surface modification steps to achieve the hydrophobicity required for MD applications [110]. These post-fabrication treatments increase process complexity, reduce production throughput, and further contribute to the overall cost of ceramic membrane manufacturing.

The use of low-cost ceramic materials such as natural clays and industrial or agricultural by-products offers a promising approach to reducing membrane production costs [111]. Although the fabrication steps remain similar to those used for high-purity ceramics, the use of inexpensive raw materials can significantly reduce production costs. For example, while conventional tubular alumina membranes have a cost between 500 and 1000 USD/m^2^, those made from kaolin can be produced for as low as 130 USD/m^2^ [112]. Various low-cost precursors have been explored, including kaolin, ball clay, bentonite, fly ash, rice husk ash (RHA), and palm oil fuel ash (POFA). These materials offer advantages such as wide availability, low cost, and suitable physicochemical properties. Natural clays, in particular, are attractive due to their chemical and thermal stability, mechanical strength, and adsorption capabilities. Clay-based membranes have shown promising performance in MD, with reported pore sizes ranging from 0.3 to 16.0 µm and porosities up to 49% [113]. Khemakhem and Amar [47] developed hydrophobic membranes using Tunisian clay with an average pore size of 0.18 µm, achieving salt rejection rates up to 99% during AGMD tests. Similarly, Abd Aziz et al. [58] fabricated superhydrophobic ceramic hollow fiber membranes from ball clay, demonstrating improved performance in DCMD, with reduced wetting and fouling.

Among these materials, kaolin has been extensively studied due to its low cost and favorable processing properties. Hydrophobic hollow fiber membranes derived from kaolin have demonstrated high MD performance, with water vapor fluxes up to 17.5 kg·m^−2^·h^−1^ and arsenic removal rates approaching 100% [98]. However, kaolin’s relatively low density (~2.4 g/cm^3^) [63] limits its mechanical strength compared to conventional ceramic materials like alumina. This limitation can be addressed through thermal treatment, which induces the formation of mullite (3Al_2_O_3_·2SiO_2_), a crystalline phase with a higher density (3.11–3.26 g/cm^3^) and enhanced mechanical and thermal properties [114]. Mullite-based membranes exhibit improved resistance to thermal shock, chemical attack, and mechanical stress, making them well-suited for harsh MD environments. Twibi et al. [56], for example, fabricated hydrophobic mullite hollow fiber membranes that maintained a water vapor flux of 22.51 kg·m^−2^·h^−1^ and salt rejection of 99.99% after 20 h of continuous DCMD using a 10 g/L NaCl feed solution. Recently, increasing attention has been given to the use of waste-derived materials. By-products from coal combustion and agro-industrial processes—such as RHA, POFA, and fly ash—contain high concentrations of silica and alumina, making them promising candidates for sustainable, low-cost ceramic membranes [111]. Dong et al. [115] reported that membrane material costs using mineral or waste-based sources can range from only 2 to 130 USD/m^2^—just a fraction (1/500 to 1/4) of the price of commercial ceramic membranes. These materials often inherently contain sintering aids (e.g., Na, K, Ca, Mg), which can reduce the sintering temperature and thus lower energy costs during processing. Moreover, novel fabrication techniques such as phase inversion, centrifugal casting, and co-sintering further reduce production costs by minimizing processing steps and energy input. These strategies not only provide economic benefits but also contribute to environmental sustainability by promoting the reuse of industrial by-products and reducing ceramic membrane production’s environmental footprint.

### 5.2. Wetting and Fouling

Wetting and fouling remain among the key limitations affecting the long-term reliability and efficiency of ceramic membranes in MD systems [116]. While ceramic membranes are well known for their outstanding thermal and chemical robustness, they can still experience a loss of vapor-phase selectivity, particularly when treating surfactant-laden or highly fouling feedwaters. The build-up of organic and inorganic matter on the membrane surface can compromise the LEP and alter surface wettability, thereby creating conditions that favor liquid breakthrough. Such interfacial instabilities can disrupt vapor transport, resulting in declining flux and a marked drop in salt rejection performance [117].

**Figure 7:**
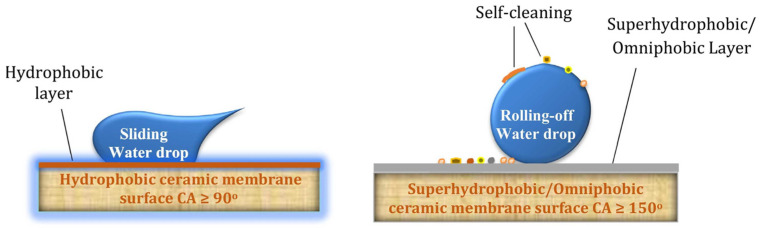
Hydrophobic and superhydrophobic ceramic membrane surfaces. Reprinted with permission from Ref. [38]. Copyright 2022 Elsevier.

A key approach to overcoming these limitations involves advanced surface engineering techniques aimed at enhancing the anti-wetting and anti-fouling properties of ceramic membranes. A widely adopted strategy is the creation of superhydrophobic surfaces (Figure 7), which integrate hierarchical surface roughness with low surface energy coatings. This configuration minimizes solid–liquid contact, encourages water droplet roll-off, and reduces foulant adhesion. For instance, ceramic membranes modified through sol–gel methods and steam impingement have achieved water contact angles above 150° and contact angle hysteresis below 10°, enabling self-cleaning behavior and improved wetting resistance [115]. Similarly, UV-assisted silane functionalization increased contact angles from 46° to nearly 159°, while maintaining stable DCMD performance with fluxes exceeding 27 kg·m^−2^·h^−1^ and salt rejection rates of 99.99% when processing saline solutions at elevated temperatures [118].

Another effective strategy is the incorporation of inorganic nanostructures on the membrane surface to enhance resistance against both organic and inorganic fouling. Ceramic membranes coated with silica–alumina nanoparticle layers achieved contact angles of approximately 158°, while delivering high fluxes (~29 L·m^−2^·h^−1^) and 99.9% salt rejection when tested in MD with 3.5 wt% NaCl feed solutions [94]. Likewise, β-sialon-based membranes reinforced with mullite whiskers exhibited contact angles up to 165° and remained operational for over 400 h under SGMD conditions using brines containing 50 ppm humic acid, without significant flux decline [92].

However, the durability of superhydrophobic surfaces can be compromised in the presence of low-surface-tension compounds such as ethanol, surfactants, or fatty acids, which may penetrate even highly water-repellent membranes [119]. To address this issue, omniphobic ceramic membranes have been developed to repel both polar and non-polar liquids by incorporating re-entrant surface geometries and fluorinated functional layers. Ceramic alumina hollow fiber membranes modified with ZnO nanostructures and fluorosilanes exhibited contact angles of 138.1° for water and 128.7° for ethanol, indicating their potential for treating organic-rich feeds [71]. Moreover, these membranes demonstrated good wetting resistance, maintaining stable permeate flux for 24 h when treating a 2.0 mM SDS solution (70 °C, 1 M NaCl) as the feed [71]. A similar strategy employing foliage-like and rod-like TiO_2_ nanostructures enabled the fabrication of omniphobic mullite membranes with contact angles of 150° for ethylene glycol and 140° for olive oil [72]. The resulting omniphobic mullite membranes significantly reduced organic fouling, even after 500 min of continuous DCMD operation using an aqueous 3.5 wt% NaCl feed solution containing 10 mg/L humic acid [72].

### 5.3. Fragility

The mechanical fragility of ceramic membranes presents a significant barrier to their integration into MD systems. This limitation originates from the intrinsic brittleness of ceramic materials, which is primarily attributed to their strong covalent and ionic bonding, as well as the presence of microstructural porosity. These characteristics result in reduced tensile and flexural strength, making ceramic membranes particularly prone to mechanical failure during fabrication, module assembly, and operation [120]. The problem is especially pronounced in hollow fiber configurations, where thin walls and small diameters amplify localized stress concentrations, increasing the risk of fracture under hydraulic pressure or turbulent flow conditions [121]. Such structural vulnerability is especially problematic for compact and modular MD units, which demand mechanically robust components capable of withstanding fluctuating thermal and hydraulic loads [120]. Structural failure not only compromises separation performance but also leads to increased maintenance frequency and operational costs, thereby limiting the scalability and economic feasibility of MD systems.

**Figure 8:**
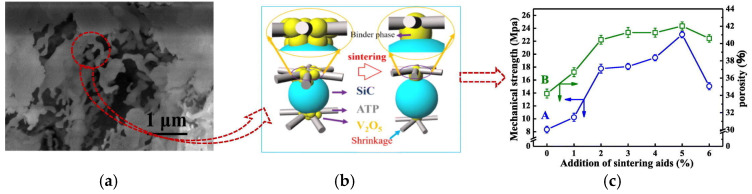
Microstructure and strengthening mechanism of porous ceramic supports prepared from low-grade palygorskite clay and SiC with V_2_O_5_ additives. (**a**) SEM image shows the porous morphology at the microscale. (**b**) The scheme illustrates the sintering process, where V_2_O_5_ acts as a liquid-phase sintering aid, promoting shrinkage and bonding among SiC and ATP particles. (**c**) The effect of sintering aid content on mechanical strength indicates an optimal additive concentration for improved performance. Copyright 2021 Elsevier. Reprinted from Ref. [124] (open access).

To address this challenge, two complementary strategies have emerged: the use of sintering additives and advanced structural design optimization. Sintering additives such as Al_2_O_3_-Y_2_O_3_, vanadium attapulgite clay, and vanadium pentoxide (V_2_O_5_) play a fundamental role in enhancing microstructural evolution during sintering. These additives facilitate liquid-phase sintering by forming transient liquid films at grain boundaries, promoting particle rearrangement, neck formation, and densification at lower temperatures. For instance, Yun et al. [122] developed a SiC membrane by incorporating Al_2_O_3_, Y_2_O_3_, and SiO_2_ as sintering additives to enhance its mechanical strength. In their study, the mixed powders were sintered at 1500 and 1600 °C for 1 h to evaluate the influence of temperature on the final properties. At 1500 °C, the membrane exhibited a substantial improvement in mechanical strength, increasing from 47.2 MPa to 71.2 MPa, while the porosity was slightly reduced from 45.9% to 42.8%. Interestingly, when the sintering temperature was raised to 1600 °C, the mechanical strength significantly decreased to approximately 32.6 MPa. This decline was attributed to excessive grain growth and densification, which led to a notable reduction in porosity and pore size, potentially compromising the balance between mechanical robustness and permeability required for membrane distillation applications [122]. Similarly, the addition of vanadium attapulgite clay has been employed to improve the crack resistance of alumina titanate ceramic membranes. This additive acts as a toughening phase by dispersing fibrous structures that hinder crack propagation pathways and enhance energy dissipation during mechanical loading. Experimental studies reported that a membrane containing 25 wt% attapulgite clay, sintered at 1300 °C, achieved a bending strength of 45.48 MPa with a maintained porosity of approximately 32%, which is critical for maintaining sufficient mass transfer in MD processes [123]. Moreover, vanadium pentoxide (V_2_O_5_) has been used as a sintering aid in ceramic supports to further enhance mechanical properties (Figure 8). Due to its low melting point, V_2_O_5_ facilitates the formation of transient liquid phases during sintering, which improves particle rearrangement and bonding. This mechanism increases membrane strength without severely reducing porosity. After sintering at 700 °C for 3 h, membranes containing V_2_O_5_ and SiC (at 34.4% content) exhibited an increase in mechanical strength to approximately 40.4 MPa, demonstrating a promising balance between structural robustness and permeability [124].

Furthermore, additional toughening mechanisms have been explored to further improve fracture resistance. One widely used strategy is the incorporation of fine particles to pin grain boundaries, a well-established mechanism that prevents excessive grain growth during sintering. By stabilizing the microstructure, these tiny particles lead to smaller and more uniformly distributed grains, which help block and deflect propagating cracks. This refined grain structure significantly enhances fracture toughness and mechanical strength, while the pinning effect reduces the likelihood of catastrophic failure by dissipating localized stress at grain interfaces. Another effective method is the YSZ (yttria-stabilized zirconia) phase transition toughening, which leverages a stress-induced phase transformation to improve crack resistance. Specifically, zirconia stabilized with yttria remains in a metastable tetragonal phase at room temperature. Under mechanical stress, this tetragonal phase transforms into a monoclinic phase, accompanied by volumetric expansion. This transformation induces compressive stresses around the crack tip, effectively hindering further crack opening and propagation. As a result, membranes reinforced with this mechanism exhibit higher fracture toughness and can withstand greater mechanical loads before failure [125]. In addition, the whisker toughening approach involves incorporating elongated, fiber-like ceramic whiskers into the membrane matrix. These whiskers act as crack-bridging elements that impede crack growth through mechanisms such as pull-out, fracture, and crack deflection. When a crack propagates, the whiskers absorb energy through frictional pull-out or by breaking, thereby dissipating mechanical energy that would otherwise drive rapid crack advancement. This mechanism not only delays crack propagation but also significantly increases the overall toughness and damage tolerance of the ceramic membrane.

In parallel, structural design strategies such as tailoring wall thickness, refining fiber geometries, and introducing graded or hierarchical porosity significantly improve mechanical robustness. Gradual pore transitions help distribute mechanical loads more evenly and reduce localized stress, thereby preventing crack initiation and propagation. The integration of reinforcing ceramic fibers within the matrix acts as a structural skeleton, maintaining integrity even if the surrounding matrix cracks [126]. Additionally, modifying the interfacial chemistry through surface coatings enhances the fiber–matrix bonding, improving pull-out resistance and overall fracture strength [121].

By strategically combining sintering aids and structural design optimization, it is possible to balance high porosity with mechanical integrity. This synergistic approach not only mitigates brittleness but also enables the fabrication of membranes that can withstand fluctuating hydraulic and thermal stresses typical in MD operations. Overall, these advancements open promising pathways toward the commercial-scale deployment of ceramic membranes for long-term, high-performance MD applications.

## 6. Emerging Applications of Ceramic Membrane in MD

Recent developments have extended the use of ceramic membranes in MD beyond conventional desalination, opening new avenues for resource recovery and sustainable water treatment. In particular, MCr and photothermal membrane distillation (PMD) have attracted increasing attention as advanced applications.

Membrane crystallization has emerged as a promising alternative technique for simultaneously producing crystals and recovering high-purity water from supersaturated solutions [20]. This approach leverages MD to concentrate the solution by selectively removing solvent in the vapor phase. In MCr systems, the membrane acts as an interface that enables solvent evaporation while controlling the final degree and rate of supersaturation generation [20,21]. As a result, MCr promotes crystal nucleation and growth through a well-controlled pathway, even starting from undersaturated solutions. Moreover, the possibility to control the transmembrane flow rate by modulating the process driving force enables precise tailoring of the final crystal properties, both in terms of structure (polymorphism) and morphology (shape, size, and size distribution) [127]. MD and MCr represent efficient strategies to enhance seawater desalination processes, particularly when integrated with RO systems. The incorporation of an MD unit to treat RO retentate can increase the overall water recovery while simultaneously reducing the volume of brine requiring disposal. Furthermore, the integration of an MCr unit offers additional advantages: it not only enhances the water recovery factor and minimizes brine discharge but also enables the production of valuable crystalline products such as NaCl [20], Na_2_SO_4_ [128], MgSO_4_·H_2_O [128], LiCl [129], etc. This dual benefit of achieving near-ZLD and recovering high-value solids underscores the significant potential of MCr in advancing sustainable and resource-efficient desalination schemes.

In this context, the high thermal and chemical stability of ceramic membranes is crucial to ensure sustained operation under supersaturated and scaling-prone conditions, thereby enabling reliable long-term MCr performance. Ko et al. [53] demonstrated the successful application of two hydrophobic alumina-based ceramic membranes in vacuum membrane crystallization (VMCr) processes using 1 M NaCl and 13 M LiCl aqueous solutions. In this study, tubular aerogel membranes (CM-L) achieved average fluxes of 1.3 L·m^−2^·h^−1^ for 1 M NaCl and 0.5 L·m^−2^·h^−1^ for 13 M LiCl solutions, while hollow fiber alumina membranes modified with fluoroalkylsilanes (CM-S) exhibited significantly higher fluxes, around 17 L·m^−2^·h^−1^ for NaCl and 3.2 L·m^−2^·h^−1^ for LiCl, without any wetting phenomena [53]. Moreover, the crystals obtained exhibited well-defined morphology and narrow size distribution, further confirming the effectiveness of ceramic membranes in enabling simultaneous high-purity water recovery and the production of high-quality crystalline products, even under challenging operating conditions.

**Figure 9:**
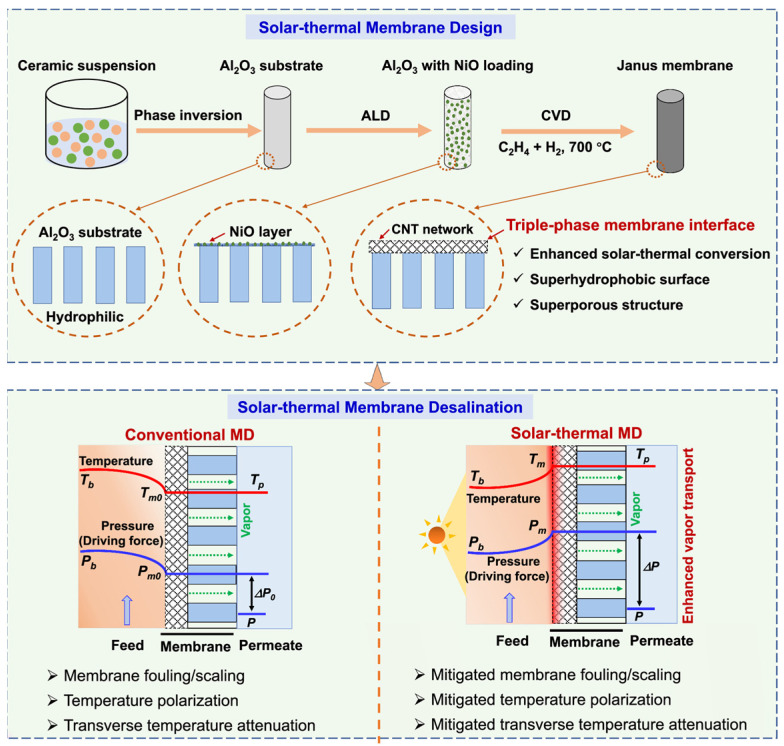
Schematic illustration of a ceramic–carbon Janus solar-thermal membrane for enhanced MD. Copyright 2025 Nature. Reprinted from Ref. [134] (open access).

Another emerging direction is PMD, a solar-assisted thermal process that integrates light-absorbing photothermal materials into membrane systems to effectively harness solar energy [130]. In this approach, solar energy is converted via photothermal reactions, enabling localized and highly efficient heat generation directly at the membrane surface, thereby facilitating sustainable vapor production in MD. The development of PMD is motivated by the necessity to address critical limitations inherent to conventional MD processes, particularly the phenomenon of temperature polarization. As described previously, in conventional MD configurations, a temperature drop occurs at the membrane-feed interface due to the formation of a thermal boundary layer [20]. This effect significantly reduces the effective vapor pressure gradient across the membrane, decreasing the driving force for mass transfer and consequently lowering water flux. Furthermore, the requirement to heat the entire bulk feed solution results in considerable thermal energy losses and compromises the overall energy efficiency of the process. PMD could overcome these challenges by enabling direct solar-to-thermal conversion at the membrane surface [130]. The integration of nanostructured photothermal materials—such as plasmonic nanoparticles, carbon-based nanomaterials, inorganic semiconductors, and polymers—promotes broadband solar absorption and facilitates rapid, localized heating [131,132]. This strategy effectively mitigates temperature polarization, maintains elevated surface temperatures, and enhances the vapor pressure gradient, leading to significantly improved water flux and overall process efficiency, thereby making PMD particularly promising for decentralized and off-grid applications and supporting sustainable, resilient water production through renewable energy utilization [133]. While most current PMD research has focused on polymeric membranes, ceramic counterparts are gaining attention due to their superior durability under high-temperature solar exposure. A recent investigation reported the development of a ceramic–carbon Janus membrane for solar-driven distillation (Figure 9), achieving solar–thermal efficiencies of 66.8–68.8% and water fluxes ranging from 3.3 to 5.1 L·m^−2^·h^−1^ under natural sunlight conditions [134]. The enhanced performance was attributed to localized surface heating, which reduced temperature polarization and increased the vapor pressure gradient across the membrane. Moreover, photothermal activation altered water structure by increasing the proportion of intermediate water and weakening hydrogen bonding, thereby lowering the enthalpy of vaporization and facilitating more efficient phase change. Transport through the membrane occurred via a combination of Knudsen diffusion and viscous flow, while stable operation in hypersaline conditions confirmed the membrane’s robustness for high-salinity, solar-powered PMD applications.

These developments highlight the potential of ceramic membranes to enable advanced, energy-efficient, and resource-recovery-focused water treatment systems.

## 7. Conclusions and Outlook

Ceramic membranes are gaining increasing recognition in MD due to their intrinsic resistance to thermal and chemical degradation, making them well-suited for the treatment of hypersaline and chemically aggressive wastewaters. Compared to conventional polymeric membranes, ceramic membranes offer enhanced durability and stability, which are crucial under harsh operational conditions. Nevertheless, their implementation at industrial scale remains constrained by high production costs, mechanical fragility, and wetting/fouling on the membrane surface.

Recent developments in the design and surface engineering of ceramic membranes—such as the integration of hierarchical surface structures, low-surface-energy coatings, and nanocomposite layers—have considerably improved their performance. The use of low-cost precursors (e.g., kaolin, fly ash, rice husk ash) further contributes to the economic sustainability of these materials. Despite these advances, the scalability of surface treatments, the long-term stability under real wastewater conditions, and the trade-off between mechanical strength and mass transport efficiency remain active areas of investigation.

Moreover, the expanding role of ceramic membranes in emerging MD configurations—such as MCr and PMD—is opening new avenues for integrated water resource recovery systems powered by renewable energy. The combination of ceramic materials with photothermal nanostructures may further enhance energy efficiency and process robustness, addressing key bottlenecks such as temperature polarization and low flux. However, ensuring material stability, membrane integrity, and environmental compatibility under long-term operation is essential for the practical implementation of ceramic MD systems at an industrial scale.

In this context, a comprehensive understanding of the interplay between membrane architecture, surface chemistry, and operational parameters is crucial for optimizing performance and advancing ceramic-based MD toward commercial viability. These developments highlight the relevance of ceramic membranes as promising candidates for advanced desalination and wastewater treatment processes, supporting sustainable water management and resource recovery strategies.

## Figures and Tables

**Table 1:** Comparative overview of the main advantages and disadvantages of the four basic MD configurations. Data extracted from [24].

MD Configuration	Advantages	Disadvantages
DCMD: the permeate side consists of a condensing fluid that is directly in contact with the membrane	- Simple and cost-effective design - The permeate is condensed inside the membrane module - Moderate transmembrane flux	- High thermal conductivity loss
AGMD: the permeate side of the membrane consists of a condensing surface separated from the membrane by an air gap	- The permeate is condensed inside the membrane module - Low thermal conductive loss with respect to DCMD	- Low transmembrane flux due to additional mass transfer resistance - More complex design compared to DCMD
SGMD: an inert sweep gas, which collects the vapor, flows at the permeate side	- Low thermal conductive loss - Moderate transmembrane flux	- The permeate is condensed outside the membrane module
VMD: a vacuum is applied at the permeate side	- Very low thermal conductive loss - High transmembrane flux	- Higher energy consumption due to vacuum - The permeate is condensed outside the membrane module - High sensitivity to membrane wetting

**Table 3:** Hydrophobization techniques for ceramic membranes in MD.

Technique	Principle	Materials/Modifiers	Advantages	Disanvantages
Chemical grafting	Covalent bonding of silane agents to surface –OH groups on ceramic membranes	Fluorosilanes (e.g., PFOTES, FAS), Methyltrimethoxysilane (MTS), hexadecyltrimethoxysilane (C16), Octyltrichlorosilane (C8)	- Good chemical and thermal stability - Tunable contact angles	- Use of fluorinated compounds - Environmental concerns
Physical deposition	Application of polymeric or nanoparticle coatings via physical methods	PDMS, PTFE, PVDF-HFP, ZnO, SiO_2_	- Simple and scalable method - Good coating uniformity	- Poor adhesion on ceramic substrates - Risk of delamination
Hierarchical structuring	Creation of micro/nano-scale surface roughness for superhydrophobicity	ZnO nanorods, TiO_2_ microflowers, Al_2_O_3_ nanoflowers	- Superhydrophobicity - Improved LEP	- Complex synthesis - Mechanical fragility
Fluorine-free coatings	Use of environmentally friendly hydrophobizing agents	Methylphenyl silicone resin, boron nitride, polydimethylsiloxane (PDMS)	- Eco-friendly alternative - Good compatibility	- Long-term stability still under investigation
Multistep modification	Combination of surface texturing and chemical grafting for enhanced performance	CuO flakes + silane; Al_2_O_3_ nanoflowers + silane	- High wetting resistance and LEP - Improved durability	- Higher fabrication cost - More complex process

## Data Availability

Data sharing is not applicable. No new data were created or analyzed in this study.
